# A factor analytic comparison of three commonly used depression scales (HAMD, MADRS, BDI) in a large sample of depressed inpatients

**DOI:** 10.1186/s12888-023-05038-7

**Published:** 2023-07-28

**Authors:** Florian Seemüller, Rebecca Schennach, Richard Musil, Michael Obermeier, Mazda Adli, Michael Bauer, Peter Brieger, Gerd Laux, Wolfgang Gaebel, Peter Falkai, Michael Riedel, Hans-Jürgen Möller

**Affiliations:** 1 Department of Psychiatry and Psychotherapy, University Hospital, Ludwig-Maximilians-University, Nussbaumstrasse 7, 80336 Munich, Germany; 2 Department of Psychiatry, Psychotherapy and Psychosomatics, Kbo-Lech-Mangfall-Klinik, Auenstrasse 6, 82467 Garmisch-Partenkirchen, Germany; 3 Schoen Clinic Roseneck, Am Roseneck 6, 83209 Prien am Chiemsee, Germany; 4 Department of Psychiatry and Psychotherapy, Charité – Universitätsmedizin Berlin, Charité Mitte (CCM), CampusCharitéplatz 1, 10117 Berlin, Germany; 5 Center for Psychiatry, Psychotherapy and Psychosomatic Medicine, Fliedner Klinik Berlin, Markgrafenstrasse 34, 10117 Berlin, Germany; 6 Department of Psychiatry and Psychotherapy, Carl Gustav Carus University Hospital Dresden, Technische Universität Dresden, Fetscherstrasse 74, 01307 Dresden, Germany; 7 Department of Psychiatry and Psychotherapy, Kbo-Isar-Amper-Klinikum Region Munich, Vockestrasse 72, 85540 Haar, Germany; 8 Institute of Psychological Medicine (IPM), Nussbaumstrasse 9, 83564 Soyen, Germany; 9 Department of Psychiatry and Psychotherapy, University of Düsseldorf, Bergische Landstrasse 2, 40629 Düsseldorf, Germany; 10 Centre for Disturbance of Memory and Demetia, Marion von Tessin Memory-Centre, Nymphenburgerstrasse 45, 80636 Munich, Germany

**Keywords:** BDI, HAMD, MADRS, Self-report, Clinician rating, Exploratory factor analysis, EFA, Confirmatory factor analysis, CFA, Factor structure, Rating scale

## Abstract

**Background:**

Quantifying depression mainly relies on the use of depression scales, and understanding their factor structure is crucial for evaluating their validity.

**Methods:**

This post-hoc analysis utilized prospectively collected data from a naturalistic study of 1014 inpatients with major depression. Confirmatory and exploratory factor analyses were performed to test the psychometric abilities of the Hamilton Depression Rating Scale, the Montgomery Asberg Depression Rating Scale, and the self-rated Beck Depression Inventory. A combined factor analysis was also conducted including all items of all scales.

**Results:**

All three scales showed good to very good internal consistency. The HAMD-17 had four factors: an "anxiety" factor, a "depression" factor, an "insomnia" factor, and a "somatic" factor. The MADRS also had four factors: a “sadness” factor, a neurovegetative factor, a “detachment” factor and a “negative thoughts” factor, while the BDI had three factors: a "negative attitude towards self" factor, a "performance impairment" factor, and a "somatic" factor. The combined factor analysis suggested that self-ratings might reflect a distinct illness dimension within major depression.

**Conclusions:**

The factors obtained in this study are comparable to those found in previous research. Self and clinician ratings are complementary and not redundant, highlighting the importance of using multiple measures to quantify depression.

## Introduction

The use of depression rating scales is essential for quantifying treatment effects [1]. Among the most established rating scales are the Hamilton Depression Rating Scale in its 17-item version (HAMD), the Montgomery Asberg Depression Rating Scale (MARDS) [2] and the Beck Depression Inventory (BDI) [3]. The HAMD was primarily developed for inpatients with major depression and includes depressive, anxious and psychovegetative symptoms. Therefore, it can detect symptoms of anxious and melancholic depression, as well as treatment effects of differential (e.g. sedative) acting antidepressants [4].

The MADRS [2] is increasingly replacing the HAMD in the context of psychopharmacological studies, and it has 10 items specifically designed for detecting symptom change in treatment trials. However, it cannot detect changes in specific symptom domains and may underestimate depression severity in subtypes [4, 5]. The BDI has achieved wide acceptance in the research community [6]. Its first version includes many cognitive symptoms and, therefore, better detects effects of cognitive psychotherapy [4]. Although self-report scales are less time-consuming, they are open to patient bias and may show smaller effect sizes in treatment trials [4].

Studies of the factor structure of commonly used depression scales are important because they can provide insights into the validity of scale scores. Factor analyses allow an examination of the covariation among the observed scale items to gain information on the latent constructs (= factors) that underlie them. There are two general types of factor analysis: exploratory factor analysis (EFA) and confirmatory factor analysis (CFA). In brief, EFA is used when the relation between the underlying factors and the observed variables is unknown and it is a hypothesis-generating approach. By contrast, CFA is a data driven approach and is appropriate when there is already some knowledge of the underlying factor structure [7].

The factor structure of all three scales has extensively been studied mainly via EFA, in outpatient samples and in populations of randomized controlled antidepressant trials. Shafer and co-workers published a meta-analysis of exploratory factor analyses including the HAMD and the BDI in 2006 [8], where a four-factor structure including an anxiety, a general depression, a sleep, and a somatic complaints factor, appeared to be the most generalizable solution for the HAMD. For the BDI a three-factor solution comprised a negative attitude towards self, a performance impairment and a somatic concerns factor [8]. As of April 2023, there seems to be no comparable meta-analysis for the MADRS. Previous research suggests either a 2 [9], a 3 [10–16] or a 4 factor solution [17–19]. However, an approximately comparable number finds a 1-factor solution most appropriate [20–28]. The most recent and comprehensive MADRS factor analysis revealed a four-factor solution that remained invariant over time [29]. Since in outpatient populations specific symptoms might occur rarely (e.g., suicidality, impaired illness insight) prior factor analyses might be biased towards a more mildly depressed patient sample [30]. For example, in the large STAR*D study on outpatients the mean HAMD score was 19.9 as compared to a mean HAMD score of 22.3 in the present study [31, 32]. Therefore, our first aim was to confirm the previously found factor structures by CFA within a large sample of inpatients with acute major depression.

Our second aim targets a long debate concerning self-administered versus observer rating scales [4]. It is still under debate whether self-report and clinician-rated depression scales measure the same or different dimensions of depression. Shafer has suggested that combining multiple depression scales is likely to measure most of the major specific domains of depression [8]. In line with this, we included all three scales in our analysis to gain insight into the dimensionality of self and clinician ratings. Despite significant symptom/item overlap between self and clinician reports, we hypothesized that most self-report items would appear on a separate “self” factor. To achieve these aims, we analysed a large dataset from a naturalistic study of depressed inpatients, with the specific goals of:

1. Confirming the specific psychometric properties of the HAMD, MADRS, and BDI within an inpatient sample, and
2. elucidating the relationship of self- vs. observer rated depression scales within an overall factor analysis.

## Method

### Sample and data collection

The main objective and details of the study protocol are described in detail elsewhere [32]. In brief, data from a large prospective, naturalistic, multicenter study (*N* = 1014) were analyzed. The study was part of the German research network, funded by the German Federal Ministry of Education and Research (BMBF). Subjects were recruited from seven German psychiatric university or research hospitals (two in Munich, two in Berlin, Tübingen, Düsseldorf, Halle) and five psychiatric district hospitals (Munich, Gabersee, and three in Berlin).

The core of this multicenter study was the biweekly observation of inpatients with a major depressive episode under naturalistic treatment conditions until discharge and a subsequent annual follow-up for a period of 4-years. These methods were described in detail in a study protocol, which allowed post-hoc analyses and which was approved by the Ethics Review Committee. Here, only data of the acute inpatient treatment period were analyzed.

Inclusion criteria were:

A) Age between 18 and 65
B) Signed written informed consent
C) Hospitalization and fulfilling of ICD-10 diagnostic criteria for any major depressive episode (ICD-10: F31.3x–5x, F32, F33, F34, F38) or for a depressive disorder not otherwise specified (ICD-10: F39) as primary diagnosis [33].

Exclusion criteria were:

A) Organic cause of depression
B) Insufficient knowledge of German language
C) Distance from place of residence to the study center of more than 100 km

Moreover, for confirmation of the diagnose of a depressive spectrum disorder according to DSM-IV as well as for the detection of relevant axis I and axis II comorbidities, the Structured Clinical Interview for DSM-IV (SCID-I and SCID-II) was used [34].

### Rating scales

Psychopathological symptoms were assessed using the Hamilton Depression Rating Scale (HAMD-17) [35]. The HAMD is a 17-item clinician rated scale that captures the severity of depression. Nine of its items can be rated on a Likert scale from 0–4 (depressed mood, suicide, work and interests, depressive retardation, excitement, anxiety-psychic, anxiety-somatic, hypochondriasis, illness insight) and 8 items on a 0–2 Likert scale (insomnia early, insomnia middle, insomnia late, appetite, somatic symptoms, genital symptoms, weight loss, illness insight). Higher values indicate higher symptom load. The German 17-item version has shown good reliability with a Cronbach’s α ranging from 0.72–0.83 [35, 36].

The Montgomery Asberg Depression Rating Scale (MADRS) is a 10-item clinician rated scale. Measures are rated on a 0–4 scale, higher values indicating more severe symptoms. The German translation has been shown to have a high internal consistency (Cornbachs α = 0.86) and a high sensitivity for change [37]. Its validity has been demonstrated by moderate to good correlations with the 17-item German version of the HAMD ranging from 0.51 to 0.89 [37].

The Beck Depression inventory is a 21-item self-report scale for depression severity. Ratings of depressive symptoms are made on a scale ranging from 0–4. Higher scores are indicative of higher symptoms. The German version of the self-rated Becks Depression Inventory (BDI) has a similar internal consistency (Cornbachs α = 0.86), good correlations with the self-rated Zung Depression scale and moderate to poor correlations with the HAMD (Pearson correlation = 0.37) [38].

All ratings were assessed by clinicians who had undergone a minimum of four years’ clinical training in psychiatry. All ratings for each patient were assessed by the same clinician. Patients were rated according to the protocol at baseline and every two weeks until discharge.

Patients were included in the analysis if at least two assessments were available.

### Treatment

Patients were treated at the discretion of the psychiatrist in charge under consideration of the international clinical guidelines for the treatment of depression (APA, WSFBP, DGPPN) [39–41]. In addition, the medication class, their active compounds, the dosage, and the treatment duration were recorded. Furthermore, the duration and type of other biological treatments like electroconvulsive treatment, sleep deprivation, transcranial magnetic stimulation and psychotherapy were carefully recorded. Detailed description of the treatment can be found elsewhere [32].

### Statistical analyses

#### Included assessments

Due to the naturalistic design the inpatient treatment time varied and each patient had a different number of visits. Usually, only a single time point is included when investigating the psychometrics of scales. In depression research, baseline ratings at study entry usually include more severe ratings with less symptom variability. However, endpoint ratings may include less severe ratings with higher variability and a bias towards a more treatment resistant population. To ensure that every patient entered the analysis with the same weight and to avoid treatment effects confounding results of the factor analysis, we used the method purposed by Uher et al. (2008) [28]. This method involves using a “random week dataset”, whereby.

one single visit was randomly chosen for each patient.

### Classical test theory

From classical test theory Cronbach’s alpha, coefficient omega and point biserial correlations were calculated to quantify the reliability of each scales. The Correlations between scales were assessed using Pearson's correlation coefficient.

### Factor structure

We chose a two-stage procedure. In the first step, a confirmatory factor analysis (CFA) was performed based on the factors found in the literature (as described in the introduction). If the CFA showed a poor model fit, a second exploratory factor analysis was performed.

### Confirmatory factor analyses (CFA)

To perform CFA of the HAMD we used the fourfactor solution found by the EFA factor metanalysis conducted by Shafer et al. [8]. This solution included an anxiety factor (HAMD-items: anxiety psychic, agitation, anxiety somatic, hypochondriasis, insight loss), depression factor (retardation, depressed mood, suicide, work and interests, guilt), insomnia factor (initial, middle, delayed) and a somatic factor (gastrointestinal, general somatic, weight loss, libido).

For the CFA of the MADRS we utilized the fourfactor solution as proposed by Quilty and others (2013) [29]. This solution included a sadness factor (apparent sadness, reported sadness), a neurovegetative factor (inner tension, reduced sleep, reduced ppetite), a detachment factor (concentration, lassitude, inability to feel) and a negative thoughts factor (pessimistic thoughts, suicidal thoughts).

For the CFA of the BDI we relied on the threefactor solution found by the metanalysis conducted by Shafer et al. (2006) [8]. This solution included a negative towards self-factor (BDI items: self-hate, sense of failure, guilt feeling, self-accusation, sense of punishment, suicidal ideas, pessimism, body image, sadness, lack of satisfaction, crying spells), a performance impairment factor (fatigue, difficulty working, social withdrawal, irritability, somatic concern, libido loss, indecisiveness), a somatic symptoms factor (appetite change, weight loss, insomnia).

For the CFA of the combined analysis we used the three factor structure published by Uher et al. (2008) [28]. This solution consists of an observed mood factor (MADRS Items: mood observed, mood reported, tension, concentration, lassitude, inability to feel; HAMD items: mood, activity, retardation, agitation, anxiety psychic, anxiety somatic, somatic symptoms, hypochondriasis; BDI item: health worry), a cognitive factor (MARDS items: pessimism, suicide; HAMD items: guilt suicide, BDI itmes: sadness, future, failure, enjoyment, guilt, punished, disappointed, blame self, suicide, crying, irritable, interest in people, decisions, ugly, work, tired) and a neurovegetative factor (MADRS items: sleep, appetite; HAMD items: insomia early insomnia middle, insomnia late, appetite, sexual, weight loss; BDI Items: sleep, appetite, weight loss, sexual interest).

The R package lavaan was used for application of confirmatory factor analysis (package version 0.6.11, R-Version 4.0.4). The provided results include an overall p-value of the factor model as well as three measures for the model fit: Tucker-Lewis index (TLI; good fi t ≥ 0.9), comparative fit index (CFI; good fi t ≥ 0.9) and the root mean square error of approximation (RMSEA; good fi t < 0.05; 0.05 ≤ reasonable fit ≤ 0.08) (CFI, TLI and RSMEA).

### Exploratory factor analysis (EFA)

For the combined analysis, we conducted an exploratory principal component factor analysis using Pearson correlation matrices. To determine a meaningful number of factors, we used parallel plots, as this method has been shown to be superior to other methods, such as the commonly used eigenvalue-greater-than-one rule.

To conduct the parallel analysis, we compared the eigenvalues of the original dataset to the averaged eigenvalues from 500 random permutations of the data. Eigenvalues greater than those of the random permutations suggest the presence of an underlying internal data structure and thus interpretable factors. We performed an oblique PROMAX rotation because dimensions of depression are expected to be correlated. To provide a clear arrangement of the results, only loadings with an absolute value greater than 0.4 were presented.

All statistical analyses were performed using the statistical software package R-Version 4.0.4

## Results

### Patients

Out of the original dataset of 1079, 59 patients had missing baseline data, resulting in 1014 patients with complete HAMD ratings, 990 complete BDI ratings, and 919 complete MADRS ratings. Therefore, for the final analysis, data on 3690 visits of 755 patients with complete data on all three scales were available. The mean number of assessments was 3.89 ± 2.9, and the mean inpatient treatment duration was 53.6 ± 47.5 days. The patients had a mean age of 45.5 ± 11.9 years, and 62.2% of the sample consisted of female patients.

### Treatment

Data regarding medication were available for 859 patients in the sample. A detailed description of the medication and prescription patterns is published elsewhere [32]. In brief, 97% of the patients received antidepressant medication either as monotherapy or in combination with other medication. Benzodiazepines were received by 58% of the patients, and 43% were prescribed hypnotics. Antipsychotic medication was taken by 44% of the patients. The ten most frequently prescribed antidepressants, in declining order, were venlafaxine (37%), mirtazapine (23%), sertraline (18%), citalopram (16%), trimipramine (15%), amitriptyline (13%), reboxetine (9%), doxepin (7%), paroxetine (5%), and tranylcypromine (5%).

### Correlations

The basic psychometrics can be found in Table 1. We found good reliability for the MADRS (Cronbach’s α and coefficient Omega = 0.92), the HAMD-17 (Cronbach’s α = 0.85, coefficient Omega = 0.86), and very good internal consistency for the BDI (Cronbach’s α = 0.91, coefficient Omega = 0.92). As expected, there was a strong correlation between the two observer scales (0.88), but a notably weaker correlation between the self-rated BDI and one of the observer rating scales (HAMD-17: 0.58 and MADRS: 0.59).

**Table 1:** Sum score correlations, mean sum scores of the random week set of HAMD-17, MADRS and BDI and internal consistency (Cronbach´s alpha, coefficient Omega)

	**MADRS**	**HAMD-17**	**Min**	**Max**	**Mean**	**SD**	**Cronbach´s alpha**	**Omega**
MADRS			1.00	51.00	22.70	10.47	0.92	0.92
HAMD-17	0.88		1.00	36.00	16.22	7.72	0.85	0.86
BDI	0.59	0.58	0.00	58.00	19.97	11.42	0.91	0.92

The correlations between single items and the total HAMD-17 score revealed only weak correlations of 0.31 for agitation (item 9) and 0.18 for illness insight (item 17) (Table 2). The correlations between single items and the total MADRS score showed good to moderate values, ranging from 0.57 (reduced sleep) to 0.85 (reported sadness) (Table 3). The correlations between single items and the BDI total score suggested weak correlations of 0.24 for the BDI item "weight loss" and 0.41 for "health anxiety," with moderate correlations across all other items, ranging from 0.49 (sleep) to 0.75 (sadness) (Table 4).

**Table 2:** Single item correlation with HAMD-17 total score and internal consistency (Cronbach’s alpha, coefficient Omega)

HAMD Item	Correlation
1-Depressed mood	0.71
2-Feelings of guilt	0.53
3-Suicide	0.48
4-Insomnia early	0.40
5-Insomnia middle	0.45
6-Insomnia late	0.46
7-Work and activities	0.67
8-Retardation	0.44
9-Agitation	0.31
10-Psychic anxiety	0.55
11-Somatic anxiety	0.51
12-Loss of appetite	0.50
13-Somatic symptoms	0.47
14-Genital symptoms	0.41
15-Hypochondrias	0.34
16-Loss of weight	0.40
17-Insight	0.18
C’s alpha	0.85
Omega	0.86

**Table 3:** Single item correlation with MADRS total score and internal consistency (Cronbach’s alpha, coefficient Omega)

MDARS item	Correlation
1-Apparent sadness	0.81
2-Reported sadness	0.85
3-Inner tension	0.59
4-Reduced sleep	0.57
5-Reduced appetite	0.59
6-Concentration	0.67
7-Lassitude	0.77
8-Inhability to feel	0.81
9-Pessimistic thoughts	0.69
10-Suicidal thoughts	0.60
C’s alpha	0.92
Omega	0.92

**Table 4:** Single item correlation with BDI total score and internal consistency (Cronbach’s alpha, coefficient Omega)

BDI Item	Correlation
BDI A-Sadness	0.75
BDI B-Future pessimism	0.69
BDI C-Feeling like failure	0.66
BDI D-Lack of enjoyment	0.71
BDI E-Guilt	0.59
BDI F-Feelings beeing punished	0.50
BDI G-Dissapointment oneself	0.67
BDI H-Self blame	0.64
BDI I-Suicidal thoughts	0.59
BDI J-Crying	0.55
BDI K-Irritability	0.52
BDI L-Interest in people	0.66
BDI M-Making decisions	0.71
BDI N-Appearence	0.53
BDI O-Work	0.68
BDI P-Sleep	0.49
BDI Q-Tiredness	0.62
BDI R-Appetite	0.54
BDI S-Weight loss	0.24
BDI T-Health anxiety	0.41
BDI U-Interest in sex	0.49
C’s alpha	0.91
Omega	0.92

### Confirmatory Factor Analysis (CFA)

#### HAMD

The CFA of the HAMD using the four-factor structure (anxiety, depression, insomnia, somatic) found by Shafer et al. (2006) [8] revealed moderate to good fit based on the CFI (0.89) and TLI criteria (0.86) and reasonable fit based on the RMSEA criterion (0.065) (Table 5).

**Table 5:** Confirmatory factor analysis (CFA) for HAMD, MADRS, BDI and the combined analysis. Tucker-Lewis index (TLI; good fi t ≥ 0.9), comparative fit index (CFI; good fit ≥ 0.9) and the root mean square error of approximation (RMSEA; good fit < 0.05; 0.05 ≤ reasonable fit ≤ 0.08)

Scale	Reference	Patients eligible	Factor number	*p*-value	CFI	TLI	RSMEA
HAMD	Shafer et al. 2006	1014	4	< 0.0001	0,89	0,86	0,065
MADRS	Quilty et al. 2013	821	4	< 0.0001	0,97	0,96	0,072
BDI	Shafer et al. 2006	990	3	< 0.0001	0,91	0,90	0,068
All scales	Uher et al. 2007	755	3	< 0.0001	0,68	0,67	0,087

#### MADRS

For the CFA of the MADRS, we utilized the recently suggested fourfactor solution (as described in the introduction), which showed good fit based on the CFI (0.97) and TLI (0.92) and good fit based on the RMSEA criterion (0.072) (Table 5).

#### BDI

The CFA of the BDI using the three-factor structure (negative self-perception, performance impairment, and somatic symptoms) found by Shafer et al. (2006) [8] revealed good fit based on both the CFI (0.91) and TLI criteria (0.90), and a good fit based on the RMSEA criterion (0.068) (as shown in Table 5).

### Combined CFA

The combined analysis using the factors found by Uher et al. (2008) resulted in poor model fit across all measures (see Table 5). As with the MADRS, we chose to further explore the factor structure using an EFA.

### Exploratory Factor Analysis (EFA)

#### Factor eigenvalues and parallel plots

**Fig. 1:**
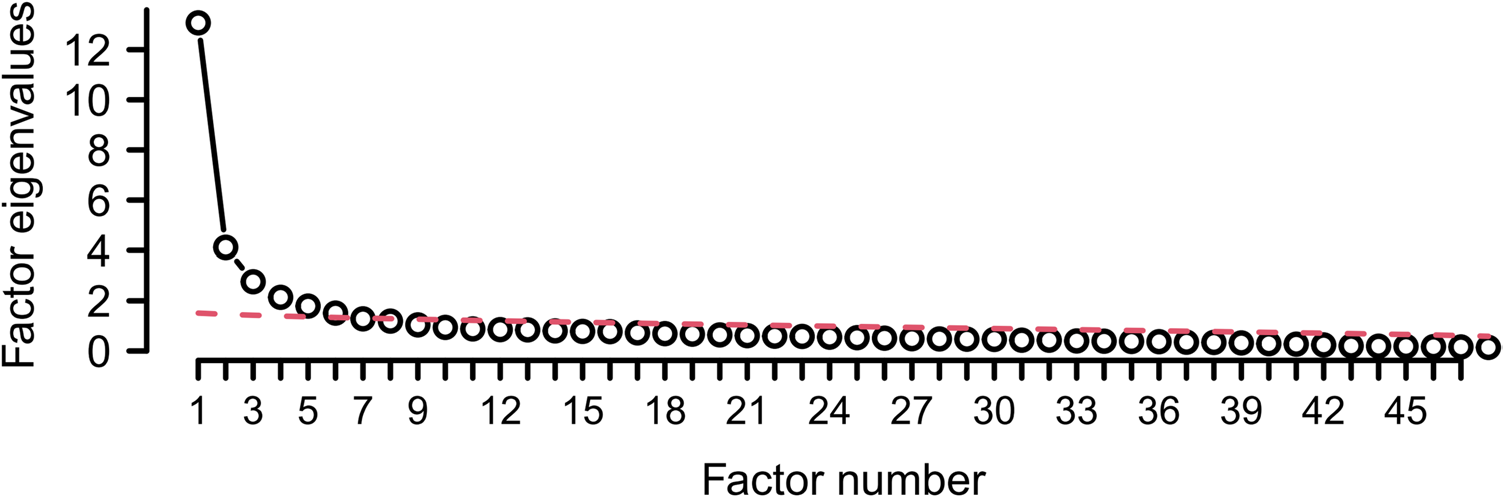
Eigenvalues of exploratory factor analysis (empty dots) compared with parallel analysis (broken line) for estimation of the number of factors for the combined EFA of all three scales (HAMD, MADRS, BDI)

The parallel analysis for the combination of all three scales suggested a 3–5-factor structure (Fig. 1). However, the difference between the simulated and observed eigenvalues decreased starting from the fourth to the sixth factor. To obtain an interpretable description and avoid too many cross-loadings, we chose a limit of three factors for the combined analysis (see discussion).

### Exploratory factor analysis of HAMD-17, MADRS and BDI

To test the hypothesis that all three scales capture one single underlying construct of depression, we conducted an EFA using all 48 items.

The first factor might be best referred to as “mood and anxiety” including observed and reported mood, genital, and somatic symptoms (HAMD) and anxiety related items on MADRS and HAM-17. The second self-rating factor exclusively consisted of BDI Items. The third “neurovegetative” factor included sleep disturbances, appetite and weight changes of all three scales and both clinician-rated suicide items (Table 6).

**Table 6:** Combined exploratory factor analysis of MADRS, BDI and HAMD, single loadings above 0.4 in a three-factor solution, single factor loadings, explained variance and internal consistency (Cronbach’s alpha)

Item	Factor 1	Factor 2	Factor 3
MADRS 1-Apparent sadness	-0.64		
MADRS 2-Reported sadness	-0.63		
MADRS 3-Inner tension	-0.63		
MADRS 4-Reduced sleep			0.73
MADRS 5-Reduced appetite			0.74
MADRS 6-Concentration	-0.73		
MADRS 7-Lassitude	-0.77		
MADRS 8-Inhability to feel	-0.72		
MADRS 9-Pessimistic thoughts	-0.68		
MADRS 10-Suicidal thoughts			
HAMD 1-Depressed mood	-0.55		
HAMD 2-Feelings of guilt	-0.51		
HAMD 3-Suicide			
HAMD 4-Insomnia early			0.60
HAMD 5-Insomnia middle			0.66
HAMD 6-Insomnia late			0.65
HAMD 7-Work and activities	-0.68		
HAMD 8-Retardation	-0.68		
HAMD 9-Agitation	-0.44		
HAMD 10-Psychic anxiety	-0.50		
HAMD 11-Somatic anxiety	-0.51		
HAMD 12-Loss of appetite			0.60
HAMD 13-Somatic symptoms	-0.58		
HAMD 14-Genital symptoms	-0.42		
HAMD 15-Hypochondrias	-0.46		
HAMD 16-Loss of weight			0.73
HAMD 17-Insight			
BDI A-Sadness		-0.63	
BDI B-Future pessimism		-0.70	
BDI C-Feeling like failure		-0.79	
BDI D-Lack of enjoyment		-0.70	
BDI E-Guilt		-0.74	
BDI F-Feelings beeing punished		-0.59	
BDI G-Dissapointment oneself		-0.78	
BDI H-Self blame		-0.76	
BDI I-Suicidal thoughts		-0.67	
BDI J-Crying		-0.48	
BDI K-Irritability		-0.53	
BDI L-Interest in people		-0.66	
BDI M-Making decisions		-0.67	
BDI N-Appearence		-0.58	
BDI O-Work		-0.62	
BDI P-Sleep			0.51
BDI Q-Tiredness		-0.55	
BDI R-Appetite			0.53
BDI S-Weight loss			0.65
BDI T-Health anxiety			
BDI U-Interest in sex		-0.47	
SS loadings	6.68	7.81	4.79
Proportion Var	0.14	0.16	0.10
Cumulative Var	0.14	0.30	0.40
C’s alpha (overal = 0.94)	0.91	0.91	0.84

## Discussion

Factor analysis of psychopathological rating scales can provide us with an estimate of the illness dimensions that underlie the respective rating scales. This naturalistic study included the three most used instruments for measuring depression in a large sample of inpatients, offering the opportunity for a comprehensive psychometric comparison.

### HAMD-17

The psychometric properties of the HAMD have been repeatedly investigated [8]. Consistent with previous findings, the HAMD-17 demonstrated good internal consistency (Cronbach’s alpha = 0.85, Omega 0,86).

Among the single item correlations, the items "agitation" and "insight" showed only weak correlations with the HAMD-17 total score, suggesting less relationship with other variables and thus little psychometric value. These two items have also consistently been described as having poor psychometric abilities, including low discriminative abilities, in previous investigations [27, 42, 43]. It has often been argued that the poor discriminative abilities of the "agitation" and "insight" items might be due to a less severe patient population. However, in this severely depressed inpatient sample, we were able to replicate these findings [28]. Additionally, in only 14% of all 3,690 visits, patients were rated as having some impairment of illness insight (HAMD item 17 > 0), suggesting a low overall prevalence of this item.

Thus, the four-factor solution suggested by Shafer’s meta-analysis was largely confirmed [8]. It suggests a “depression factor” with core depressive symptoms, a “sleep” factor, an “anxiety factor” and a “somatic symptoms” factor. We additionally checked all 3- and 4-factor solutions cited by Bagby et al. (2004) [42] and found no factor model with a better fit [44–48]. However, the factor structure proposed by Onega et a. (1997) [49] had almost the same factor structure containing the same items and also showed good model fit (CFI: 0.87, TLI: 0.85, RMSEA: 0.069).

The symptoms of the depression factor fit nicely into Parker's suggestion of classifying depression along psychomotor disturbances, which is the most specific symptom for melancholic depression [50]. In the aforementioned review on the HAMD scale summarizing results from 15 factor analyses on the HAMD-17, Bagby and coworkers (2004) also found good evidence for the presence of such a general “depression factor” [42].

The “anxiety” factor, included all anxiety related HAMD symptoms, in addition to “agitation”. Bagby´s review also suggested the presence of an “excitement factor” (including anxiety items along with agitation) as was found in 6 of the 15 reviewed samples [42]. In line with clinical experience, agitation in major depression is closely related to anxiety, as it might be its physiological manifestation. This notion is also supported by findings from Angst et al. (2008) who found that agitated depression was not significantly related to bipolarity but rather closely related to anxiety symptoms [51]. Maybe anxiety represents a separate dimension within depression [52, 53] and may be related to worse clinical outcomes [54–57]. But on the other hand anxiety symptoms are not very specific to depressive disorders, as anxiety symptoms are among the most prevalent psychopathological symptoms generally [58].

### MADRS

The CFA demonstrated a good model fit for all parameters (CFI: 0.975;TLI: 0.962; RSMEA 0,072). Our findings align well with the results reported by Williamson (2006), who initially proposed this four-factor solution in individuals with Bipolar-I disorder [18]. The four-factor solution comprises factors related to sadness, neurovegetative symptoms, detachment, and negative thoughts. In 2013, Quilty and colleagues successfully replicated this four-factor solution and found a good model fit (CFI: 0.92; RMSEA: 0.06). They also demonstrated the invariance of this solution over time and gender [29].

Furthermore, the authors presented support for a hierarchical model where all four factors loaded onto a second overarching depression factor. Additionally, we examined the one-factor solution as proposed by Uher and colleagues but only observed a good model fit in two out of the three indices (CFI: 0.94; TLI: 0.92; RMSEA: 0.107) [29].

The high correlation between single items and the MADRS total score highlights the scale's excellent reliability. Compared to the HAMD, the MADRS may be better suited for detecting or measuring treatment effects within a homogeneous sample, but it may have limitations in capturing different dimensions of illness.

### BDI

The CFA of the BDI using the factor structure of Shafer et al. (2006) confirmed the 3-factor solution with good model fit indices (Table 5). The three factors could probably be best referred to as the “negative perception of oneself" factor, as the “performance” factor and as the “somatic” factor [8].

First, this result should also be seen against the background of the developmental procedure of the BDI. Aron T. Beck developed this self-rating instrument based n his depression theory of the “cognitive triad”. The core of this theory is the assumption that depression arises from negative thoughts on the self, the world, and the future. Consequently, he developed a questionnaire that includes 5 cognitive items covering content of negative self-perception (feeling like failure, guilt, feeling of being punished, disappointment in oneself, self-blame).

In our CFA, all 5 items were indeed found to load on a single factor. From a methodological perspective, a single factor is more likely to emerge when an instrument contains similar items. However, it may be that negative self-perceptions play an important role, particularly within the subjective dimension of depression. Supporting this notion, suicidality had the highest loadings on this factor. In this context, suicidality may represent the most severe form of negative self-perception, where one feels so worthless that their life is not worth living. Beck himself also described a factor called "negative attitude towards self," which aligns well with the second factor found in our analysis [59].

These factors are also in good accordance with the results from the German BDI validation study conducted by Hautzinger et al. (1991) in a sample of 477 primarily (89%) inpatients diagnosed with a depressive episode according to ICD-9 [38]. Describing a 3-factor solution, the study proposed a "performance impairment factor" (including items such as work, tiredness, interest in people, sadness, making decisions, crying, and irritability), a "negative self-perception factor" (including items such as guilt, self-blame, feeling like a failure, feelings of being punished, future pessimism, and suicidal thoughts), and a "physical symptoms factor" (including items such as weight loss, sleep disturbances, and appetite loss) [38].

### Combined exploratory factor analysis of BDI, MDRS and HAMD

In line with our hypothesis, we found that a 3-factor solution was the best fitting and most interpretable. Only the BDI items related to sleep, appetite, and weight loss loaded together with similar items from the HAMD and MADRS, forming a separate “psychovegetative” factor. These symptoms are not specific to depression but are sensitive markers of depression within a correctly diagnosed depressed patient sample. All other self-rated items loaded onto one strong factor explaining 17% of the total variance of all scale items. This strongly supports the notion that self-ratings in major depression may represent a separate illness dimension. Considering that the HAMD and BDI have a 50% overlap in symptoms, the strict separation into two separate factors is remarkable. Uher et al. (2008) also performed an EFA with BDI, MADRS, and HAMD-17 items and found a 3-factor solution to be the most interpretable. They found one strong self-rating factor with almost all BDI items plus suicide (HAMD-17, MADRS) and guilt (HAMD-17), a mood and anxiety factor (MADRS and HAMD-17), and a neurovegetative factor with sleep and appetite items combined from all three scales [28].

The poor agreement between self-ratings and clinician-ratings is also reflected in the correlations of 0.58 and 0.59 between HAMD and MADRS with the BDI, respectively. Apart from the differing item content of self- and observer-rated scales, there are several reasons described in the literature that contribute to the discrepancy between self- and observer-rated scales in depression research.

First, self-ratings are more prone to be biased by depression severity. For instance, severely depressed patients tend to underestimate their symptomatology whereas less severely depressed patients may overestimate their symptoms [60–62]. Second, some aspects of psychopathology cannot be adequately assessed by self-ratings, as they are mainly observable by an observer, such as psychomotor retardation or hypochondriasis. Third, self-ratings are particularly vulnerable to fixed response biases in some patients, such as acquiescence bias, social desirability bias, or symptom exaggeration in the hope of receiving better care [63]. Fourthly, the accurate completion of a self-rating is dependent on the educational background and the patients´ ability for introspection [64].

However, clinician ratings are not without bias as they might be easily influenced by the clinician's expectations of the allocated treatment, which is especially true within naturalistic non-blinded conditions. Despite these limitations, self-rating might still represent a dimension of its own [4].

In our data, this notion is highlighted by the fact that even core depressive items that are closely connected or almost identical in content, such as reported sadness (MADRS), depressed mood (HAMD), and sadness (BDI), load on one self-rated factor (BDI) and one observer-rated factor (HAMD and MADRS) (Table 6).

### Strengths and limitations

Strengths of this analysis are the simultaneous application of the three depression scales most widely in use, the large sample size of inpatients including acutely suicidal patients and the independent funding by the German ministry for education and research.

But there are also some principle and methodological limitations which must be carefully considered.

Firstly, although many severely depressed inpatients were included, this sample may not be easily generalizable. Although we only had missing baseline data for a small number of patients (*n* = 59), we did not have data for all three scales at all time points, limiting generalizability. Additionally, the German healthcare system allows for easier access to treatment and longer inpatient treatment durations than in other countries. Further, older patients and adolescents are clearly underrepresented in our sample.

Secondly, all scales were assessed by the same clinician, which implies that one rating may have influenced the other. However, an independent rating would have required double the number of raters and increased the variance in ratings, leading to more "background noise".

Thirdly, several depression items were present on all three scales, suggesting some degree of redundancy. This overrepresentation of similar items could have hindered the emergence of more distinct and well-defined factors in the combined factor analysis of all three scales. On the other hand, this overlap allowed us to confirm the existence of a "self-rating dimension," since even very similar items loaded onto different factors.

Fourthly, the depression scales used did not include atypical depressive features such as overeating, oversleeping, or mood reactivity, which prevented exploration of an "atypical depression" factor. Atypical depression may be a distinct subtype of major depression associated with specific symptoms. In another study, we found that 15% of this sample met the criteria for atypical depression [65].

Fifthly, we chose to use a "random week dataset", which excluded observations of factor structures over time. However, our primary goal was to obtain a representative picture of the psychometric properties of the three scales. Focusing only on baseline ratings would have resulted in a dataset with less variability. Alternatively, if we had included discharge data, it would have biased our results towards a more treatment-resistant population. Galinowsky and colleagues (1995) reported a 2-factor solution at the beginning and a clear 1-factor solution for the MADRS at the end of antidepressant treatment, suggesting instability of the MADRS factors over time [26]. Factor instability has also been reported for the Inventory of Depressive Symptoms (IDS) and its short forms, as well as the HAMD, by Fried et al. (2016), and for the BDI [66]. On the other hand, Quilty and colleagues (2013) found factor invariance over time for the MADRS [29], as several other researchers have also demonstrated for the CDS [67, 68]. We therefore additionally computed CFA only for baseline and endpoint ratings for the combined factor analyses and found no substantial different results. Uher et al. (2008) also tested for invariance over time performing a longitudinal CFA. In line with our results the authors found invariance for factor one and three and only a minor deterioration in factor two (the self-report factor) [28]. However, this remains an important issue for further research.

Sixthly, we could have used more sophisticated statistical methods, such as hierarchical models to test whether the identified factors load on a single second-order factor, bifactor models to determine if both a global overall and specific first-order factors are present, or multitrait-multimethod analyses to reflect both the dimensions and rating perspectives simultaneously. However, since most of the cited research used similar methods, our results are better comparable.

### Future perspectives

Our analysis confirmed the multidimensionality of the HAMD-17 and the BDI and the the MADRS. Additionally, we observed the emergence of a distinct subjective dimension represented by the BDI. However, what are the potential consequences and implications of these findings? Symptoms of major depression may consist of clusters that are associated with distinct neurochemical disturbances [12]. For example, suicide and aggressive behaviour may be related to hypoactivity of serotonin, while psychomotor retardation and anhedonia may be related to hypoactivity of norepinephrine and dopamine [12].

A reasonable application of such results, for example, could be their use in neurobiological research. Instead of simply correlating the overall sum scores of depression scales with biological variables (e.g. serotonin binding capacities, fMRI), a more sophisticated approach could be used. Hypothesis-guided correlation of the respective depression dimension with an a priori assumed biological correlate could be a useful approach to discover new neurobiological substrates. In addition, for more detailed psychopathological analyses, such as predictive power of specific symptoms, using factors instead of forcing all variables of a rating scale into one statistical model (i.e., logistic regression) and being confronted with the problem of multicollinearity could be an alternative. In this regard, the issue of factor invariance across clinically meaningful endpoints, such as responders versus non-responders or remitters versus non-remitters, represents another crucial aspect to consider. Future analyses could further investigate hierarchical models that explore the underlying factors contributing to the construct of depression. Since clear biological measures of depression are lacking, quantifying depression and treatment effects still relies on detailed psychopathology using instruments with proven psychometric abilities. This goal is likely best reached with multiple complementary measures. This holds especially true as we are repeatedly reminded of the dimensionality of these disorders.

## Data Availability

The data that support the findings of this study are not openly available due to reasons of sensitivity and are available from the corresponding author upon reasonable request with the permission of the study centers. Data are in controlled access data storage at the Ludwig-Maximilians-University.

## References

[1] von Glischinski M, von Brachel R, Thiele C, Hirschfeld G (2021). Not sad enough for a depression trial? A systematic review of depression measures and cut points in clinical trial registrations. J Affect Disord.

[2] Montgomery SA, Asberg M (1979). A new depression scale designed to be sensitive to change. BrJ Psychiatry.

[3] Beck T, Ward H, Mendelsson M, Mok J, Erbaug J (1961). An inventory for measuring depression. Arch Gen Psychiatry.

[4] Demyttenaere K, Jaspers L (2020). Trends in (not) using scales in major depression: A categorization and clinical orientation. Eur Psychiatry.

[5] Musil R, Seemüller F, Meyer S, Spellmann I, Adli M, Bauer M, Kronmüller KT, Brieger P, Laux G, Bender W (2018). Subtypes of depression and their overlap in a naturalistic inpatient sample of major depressive disorder. Int J Methods Psychiatr Res.

[6] Schwab J, Bialow M, Clemmons R, Martin P, Holzer C (1967). The Beck depression inventory with medical inpatients. Acta PsychiatrScand.

[7] Zieminska E, Stafiej A, Lazarewicz JW (2003). Role of group I metabotropic glutamate receptors and NMDA receptors in homocysteine-evoked acute neurodegeneration of cultured cerebellar granule neurones. NeurochemInt.

[8] Shafer AB (2006). Meta-analysis of the factor structures of four depression questionnaires: Beck, CES-D, Hamilton, and Zung. J Clin Psychol.

[9] Hammond MF (1998). Rating depression severity in the elderly physically ill patient: reliability and factor structure of the Hamilton and the Montgomery-Asberg Depression Rating Scales. Int J Geriatr Psychiatry.

[10] Suzuki A, Aoshima T, Fukasawa T, Yoshida K, Higuchi H, Shimizu T, Otani K (2005). A three-factor model of the MADRS in major depressive disorder. Depress Anxiety.

[11] Ketharanathan T, Hanwella R, Weerasundera R, de Silva VA (2016). Diagnostic Validity and Factor Analysis of Montgomery-Asberg Depression Rating Scale in Parkinson Disease Population. J Geriatr Psychiatry Neurol.

[12] Parker RD, Flint EP, Bosworth HB, Pieper CF, Steffens DC (2003). A three-factor analytic model of the MADRS in geriatric depression. IntJ GeriatrPsychiatry.

[13] Gabryelewicz T, Styczynska M, Pfeffer A, Wasiak B, Barczak A, Luczywek E, Androsiuk W, Barcikowska M (2004). Prevalence of major and minor depression in elderly persons with mild cognitive impairment–MADRS factor analysis. Int J Geriatr Psychiatry.

[14] Borentain S, Gogate J, Williamson D, Carmody T, Trivedi M, Jamieson C, Cabrera P, Popova V, Wajs E, DiBernardo A (2022). Montgomery-Åsberg Depression Rating Scale factors in treatment-resistant depression at onset of treatment: Derivation, replication, and change over time during treatment with esketamine. Int J Methods Psychiatr Res.

[15] Benazzi F (2001). Factor analysis of the Montgomery Asberg Depression Rating Scale in 251 bipolar II and 306 unipolar depressed outpatients. Prog Neuropsychopharmacol Biol Psychiatry.

[16] Andersson S, Krogstad JM, Finset A (1999). Apathy and depressed mood in acquired brain damage: relationship to lesion localization and psychophysiological reactivity. Psychol Med.

[17] Basu A, Chadda R, Sood M, Rizwan SA (2017). Pre-treatment factor structures of the Montgomery and Åsberg Depression Rating scale as predictors of response to escitalopram in Indian patients with non-psychotic major depressive disorder. Asian J Psychiatr.

[18] Williamson D, Brown E, Perlis RH, Ahl J, Baker RW, Tohen M (2006). Clinical relevance of depressive symptom improvement in bipolar I depressed patients. J Affect Disord.

[19] Craighead WE, Evans DD (1996). Factor analysis of the Montgomery-Asberg Depression Rating Scale. Depression.

[20] Yee A, Yassim AR, Loh HS, Ng CG, Tan KA (2015). Psychometric evaluation of the Malay version of the Montgomery- Asberg Depression Rating Scale (MADRS-BM). BMC Psychiatry.

[21] Wolthaus JE, Dingemans PM, Schene AH, Linszen DH, Knegtering H, Holthausen EA, Cahn W, Hijman R (2000). Component structure of the positive and negative syndrome scale (PANSS) in patients with recent-onset schizophrenia and spectrum disorders. Psychopharmacology.

[22] Rocca P, Fonzo V, Ravizza L, Rocca G, Scotta M, Zanalda E, Bogetto F (2002). A comparison of paroxetine and amisulpride in the treatment of dysthymic disorder. J Affect Disord.

[23] Khan A, Khan SR, Shankles EB, Polissar NL (2002). Relative sensitivity of the Montgomery-Asberg Depression Rating Scale, the Hamilton Depression rating scale and the Clinical Global Impressions rating scale in antidepressant clinical trials. Int Clin Psychopharmacol.

[24] Hallit S, Obeid S, El Hage W, Kazour F (2019). Validation of the Arabic version of the MADRS scale among Lebanese patients with depression. Encephale.

[25] Carmody TJ, Rush AJ, Bernstein I, Warden D, Brannan S, Burnham D, Woo A, Trivedi MH (2006). The Montgomery Asberg and the Hamilton ratings of depression: a comparison of measures. Eur Neuropsychopharmacol.

[26] Galinowski A, Lehert P (1995). Structural validity of MADRS during antidepressant treatment. IntClin Psychopharmacol.

[27] Bunevicius A, Staniute M, Brozaitiene J, Pommer AM, Pop VJ, Montgomery SA, Bunevicius R (2012). Evaluation of depressive symptoms in patients with coronary artery disease using the Montgomery Åsberg Depression Rating Scale. Int Clin Psychopharmacol.

[28] Uher R, Farmer A, Maier W, Rietschel M, Hauser J, Marusic A, Mors O, Elkin A, Williamson RJ, Schmael C (2008). Measuring depression: comparison and integration of three scales in the GENDEP study. PsycholMed.

[29] Quilty LC, Robinson JJ, Rolland JP, Fruyt FD, Rouillon F, Bagby RM (2013). The structure of the Montgomery-Åsberg depression rating scale over the course of treatment for depression. Int J Methods Psychiatr Res.

[30] Seemuller F, Riedel M, Obermeier M, Bauer M, Adli M, Kronmuller K, Holsboer F, Brieger P, Laux G, Bender W (2010). Outcomes of 1014 naturalistically treated inpatients with major depressive episode. EurNeuropsychopharmacol.

[31] Rush AJ, Trivedi MH, Wisniewski SR, Nierenberg AA, Stewart JW, Warden D, Niederehe G, Thase ME, Lavori PW, Lebowitz BD (2006). Acute and longer-term outcomes in depressed outpatients requiring one or several treatment steps: a STAR*D report. AmJ Psychiatry.

[32] Seemüller F, Riedel M, Obermeier M, Bauer M, Adli M, Kronmüller K, Holsboer F, Brieger P, Laux G, Bender W (2010). Outcomes of 1014 naturalistically treated inpatients with major depressive episode. European neuropsychopharmacology: the journal of the European College of Neuropsychopharmacology.

[33] World Health O, World Health O (1992). The ICD-10 Classification of Mental and behavioural Disorders.

[34] Wittchen HU, Wunderlich U, Gruschwitz S, Zaudig M. Strukturiertes Klinisches Interview für DSM-IV. G”ttingen: Hogrefe; 1997.

[35] Baumann U (1976). Methodologic studies of the Hamilton rating scale for depression (author's transl). Arch PsychiatrNervenkr.

[36] Maier W, Philipp M, Gerken A (1985). [Dimensions of the Hamilton Depression Scale. Factor analysis studies]. EurArch Psychiatry Neurol Sci.

[37] Schmidtke A, Fleckenstein P, Moises W, Beckmann H (1988). Studies of the reliability and validity of the German version of the Montgomery-Asberg Depression Rating Scale (MADRS). SchweizArch Neurol Psychiatr.

[38] Hautzinger M (1991). The Beck Depression Inventory in clinical practice. Nervenarzt.

[39] Bauer M, Bschor T, Pfennig A, Whybrow PC, Angst J, Versiani M, Möller H-J (2007). World Federation of Societies of Biological Psychiatry (WFSBP) Guidelines for Biological Treatment of Unipolar Depressive Disorders in Primary Care. World J Biol Psychiatry.

[40] Apa: Practice guideline for the treatment of patients with major depressive disorder (revision). 2000;157:1–45. 10767867

[41] Dgppn: Practice Guidelines for the Treatment of Affective Disorders. In*.* Darmstadt: Steinkopf; 2000.

[42] Bagby RM, Ryder AG, Schuller DR, Marshall MB (2004). The Hamilton Depression Rating Scale: has the gold standard become a lead weight?. AmJ Psychiatry.

[43] Evans KR, Sills T, DeBrota DJ, Gelwicks S, Engelhardt N, Santor D (2004). An Item Response analysis of the Hamilton Depression Rating Scale using shared data from two pharmaceutical companies. J PsychiatrRes.

[44] Berrios GE, Bulbena-Villarasa A (1990). The Hamilton Depression Scale and the numerical description of the symptoms of depression. Psychopharmacol Ser.

[45] Fleck MP, Poirier-Littre MF, Guelfi JD, Bourdel MC, Loo H (1995). Factorial structure of the 17-item Hamilton Depression Rating Scale. Acta Psychiatr Scand.

[46] Marcos T, Salamero M (1990). Factor study of the Hamilton Rating Scale for Depression and the Bech Melancholia Scale. Acta Psychiatr Scand.

[47] Pancheri P, Picardi A, Pasquini M, Gaetano P, Biondi M (2002). Psychopathological dimensions of depression: a factor study of the 17-item Hamilton depression rating scale in unipolar depressed outpatients. J Affect Disord.

[48] Smouse PE, Feinberg M, Carroll BJ, Park MH, Rawson SG (1981). The Carroll rating scale for depression. II. Factor analyses of the feature profiles. Br J Psychiatry.

[49] Onega LL, Abraham IL (1997). Factor structure of the Hamilton Rating Scale for Depression in a cohort of community-dwelling elderly. Int J Geriatr Psychiatry.

[50] Parker G, Hadzi-Pavlovic D, Brodaty H, Boyce P, Mitchell P, Wilhelm K, Hickie I, Eyers K (1993). Psychomotor disturbance in depression: defining the constructs. J AffectDisord.

[51] Angst J, Gamma A, Benazzi F, Ajdacic V, Rossler W. Does psychomotor agitation in major depressive episodes indicate bipolarity? : Evidence from the Zurich Study. EurArch Psychiatry Clin Neurosci. 2008. 10.1007/s00406-008-0834-7 18806921

[52] Möller H-J, Bandelow B, Volz H-P, Barnikol UB, Seifritz E, Kasper S (2016). The relevance of ‘mixed anxiety and depression’ as a diagnostic category in clinical practice. Eur Arch Psychiatry Clin Neurosci.

[53] Bühler J, Seemüller F, Läge D (2014). The predictive power of subgroups: an empirical approach to identify depressive symptom patterns that predict response to treatment. J Affect Disord.

[54] Wiethoff K, Bauer M, Baghai TC, Moller HJ, Fisher R, Hollinde D, Kiermeir J, Hauth I, Laux G, Cordes J, et al. Prevalence and treatment outcome in anxious versus nonanxious depression: results from the German algorithm project. J Clin Psychiatry. 2010. 10.4088/JCP.09m05650blu 20673545

[55] Fava M, Rush AJ, Alpert JE, Balasubramani GK, Wisniewski SR, Carmin CN, Biggs MM, Zisook S, Leuchter A, Howland R (2008). Difference in treatment outcome in outpatients with anxious versus nonanxious depression: a STAR*D report. AmJ Psychiatry.

[56] Riedel M, Möller H-J, Obermeier M, Adli M, Bauer M, Kronmüller K, Brieger P, Laux G, Bender W, Heuser I (2011). Clinical predictors of response and remission in inpatients with depressive syndromes. J Affect Disord.

[57] Kautzky A, Möller HJ, Dold M, Bartova L, Seemüller F, Laux G, Riedel M, Gaebel W, Kasper S (2021). Combining machine learning algorithms for prediction of antidepressant treatment response. Acta Psychiatr Scand.

[58] Alpers GW (2009). Ambulatory assessment in panic disorder and specific phobia. PsycholAssess.

[59] Beck AT, Beamesderfer A. Assessment of depression: the depression inventory. In: Modern problems of pharmacopsychiatry. edn.: Karger, Basel; 1974. 10.1159/000395074 4412100

[60] Moller HJ (1991). Outcome criteria in antidepressant drug trials: self-rating versus observer-rating scales. Pharmacopsychiatry.

[61] Moller HJ, von Zerssen D (1995). Self-rating procedures in the evaluation of antidepressants. Psychopathology.

[62] Prusoff BA, Klerman GL, Paykel ES (1972). Concordance between clinical assessments and patients' self-report in depression. Arch Gen Psychiatry.

[63] Moller HJ (2000). Rating depressed patients: observer- vs self-assessment. EurPsychiatry.

[64] Demyttenaere K, Desaiah D, Petit C, Croenlein J, Brecht S (2009). Patient-Assessed Versus Physician-Assessed Disease Severity and Outcome in Patients With Nonspecific Pain Associated With Major Depressive Disorder. PrimCareCompanionJ Clin Psychiatry.

[65] Seemuller F, Riedel M, Wickelmaier F, Adli M, Mundt C, Marneros A, Laux G, Bender W, Heuser I, Zeiler J, et al. Atypical symptoms in hospitalised patients with major depressive episode: frequency, clinical characteristics, and internal validity VL. J AffectDisord. 2007. 10.1016/j.jad.2007.10.025 18164767

[66] Richter P, Werner J, Heerlein A, Kraus A, Sauer H (1998). On the validity of the Beck Depression Inventory. A review Psychopathology.

[67] Ferro MA, Speechley KN (2013). Factor structure and longitudinal invariance of the Center for Epidemiological Studies Depression Scale (CES-D) in adult women: application in a population-based sample of mothers of children with epilepsy. Arch Womens Ment Health.

[68] Zhu X, Shek DTL, Dou D (2021). Factor structure of the Chinese CES-D and invariance analyses across gender and over time among Chinese adolescents. J Affect Disord.

